# Recent Progress in Polysaccharide Aerogels: Their Synthesis, Application, and Future Outlook

**DOI:** 10.3390/polym13081347

**Published:** 2021-04-20

**Authors:** Arbanah Muhammad, Dabin Lee, Yonghun Shin, Juhyun Park

**Affiliations:** Department of Intelligent Energy and Industry, School of Chemical Engineering and Materials Science, Chung-Ang University, Seoul 06974, Korea; arbanah7188@uitm.edu.my (A.M.); imdabin@naver.com (D.L.); dydgns1289@naver.com (Y.S.)

**Keywords:** porous, polysaccharides, aerogel

## Abstract

Porous polysaccharides have recently attracted attention due to their porosity, abundance, and excellent properties such as sustainability and biocompatibility, thereby resulting in their numerous applications. Recent years have seen a rise in the number of studies on the utilization of polysaccharides such as cellulose, chitosan, chitin, and starch as aerogels due to their unique performance for the fabrication of porous structures. The present review explores recent progress in porous polysaccharides, particularly cellulose and chitosan, including their synthesis, application, and future outlook. Since the synthetic process is an important aspect of aerogel formation, particularly during the drying step, the process is reviewed in some detail, and a comparison is drawn between the supercritical CO_2_ and freeze drying processes in order to understand the aerogel formation of porous polysaccharides. Finally, the current applications of polysaccharide aerogels in drug delivery, wastewater, wound dressing, and air filtration are explored, and the limitations and outlook of the porous aerogels are discussed with respect to their future commercialization.

## 1. Introduction

**Figure 1:**
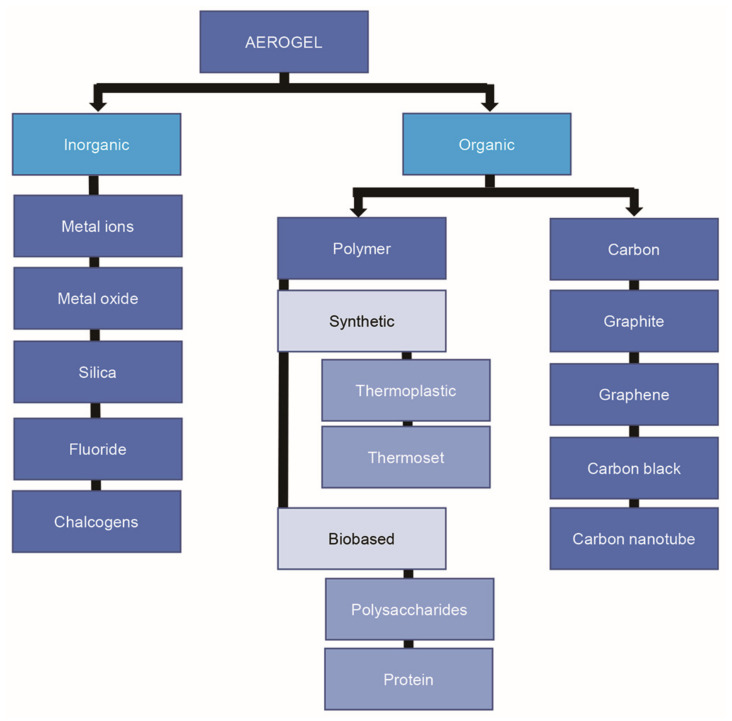
Classification of aerogels.

An aerogel is a synthetic, porous, and ultra-light material derived from a gel in which the liquid of the gel is replaced with a gas and its structure is maintained. About 99% of the aerogel is air, which makes it the lightest material on Earth [1,2,3,4,5]. Aerogel development was started in 1931 by Klister, and since then the definition of aerogel has become broader. Aerogel-forming materials can be manufactured using inorganic compounds, organic polymers obtained from polysaccharides and proteins, synthetic polymers, or a mixture of organic and synthetic polymers [1,6,7,8,9]. Hence, aerogels can be broadly classified as inorganic and organic according to their precursors, as shown schematically in Figure 1, where the polysaccharides are classified as a biobased organic polymer source. The organic polymer sources also include synthetic polymers, which are subdivided into the thermoplastic and thermoset polymers. In addition, aerogel structures can be classified according to their pore diameters as mesoporous (2–50 nm), microporous (<2 nm), or macroporous (>50 nm) [10]. As discussed in the literature, any highly porous material with a three-dimensional (3-D) network of gas, low density, and large inner surface area is considered to be an aerogel. The density of an aerogel can be as low as 0.004–0.5 g/cm^3^ with a high porosity of up to 99.8% and a large specific surface area of 100–1600 m^2^/g [11]. Due to these properties, the aerogels have been applied as adsorbents of CO_2_ [12,13,14,15,16], dyes, and heavy metals [2,3,11,17,18,19,20,21,22,23], as tissue engineering and blood system scaffolds in the biomedical field [3,22,23,24,25,26,27], and as materials for thermal insulation [4,28,29,30,31,32,33,34,35,36] and food packaging [37,38,39,40,41]. These versatile materials also exhibit high optical transparencies, low thermal conductivities (down to 12 mW/mK) and low sound speeds [42,43]. Moreover, they exhibit outstanding properties such as high strengths, high thermal isolations, and low dielectric constants, making them suitable for applications that involve heating elements [30,31].

**Figure 2:**
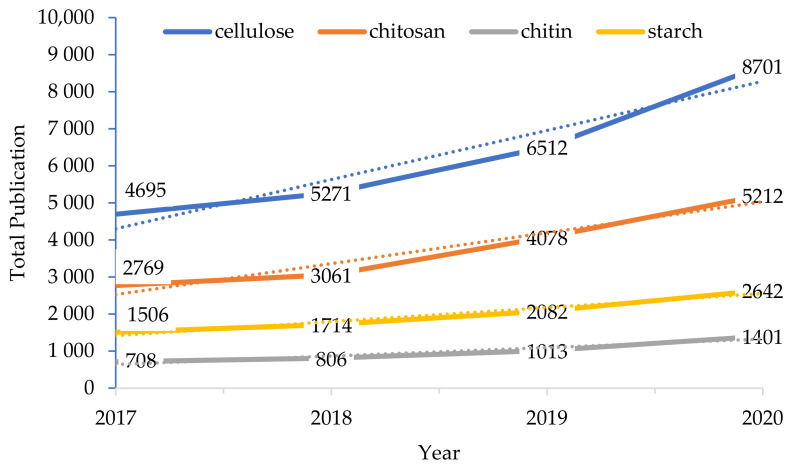
Four types of polysaccharide gel reported in the literature according to year of publication (from a search using the keyword ‘porous polysaccharides’ on Science Direct, 18 October 2020).

More recently, there has been a growing interest in the development of bio-aerogels from natural polysaccharides due to their enhanced biosafety, availability, and biodegradability compared to those of inorganic and organic chemicals [1,6,44]. These promising materials are derived from naturally occurring polymers and biopolymers, which are found abundantly in natural structural building blocks. Natural polysaccharides contain crystalline and amorphous regions that are highly susceptible to hydrolysis. Various polysaccharides can be used to produce aerogels, including cellulose [24], starch [45], alginate [46], pectin [47], lignin [29], collagen [48], and chitosan [44]; however, cellulose and chitosan/chitin are the most commonly used and have therefore attracted current research interest. This is reflected in the plot of four types of polysaccharide gel reported in the literature against the year of publication in Figure 2, where cellulose is seen to be the most reported since 2016. 

Cellulose can be derived from plant cell walls (wood) or from microorganisms (e.g., bacteria), and can take the form of nanocrystals, nanofibrils, nanocomposite, and bacterial cellulose [12,49]. Long, Weng and Wang (2018) classified cellulose aerogel into three categories, namely: natural cellulose, regenerated cellulose, and cellulose derivative aerogels [50]. Aerogels produced from plant cellulose require the extraction of impurities such as lignin, wax, pectin, and hemicellulose, a process that is also known as delignification [29]. The isolation of a nanocrystal from cellulose can be performed using chemical or enzymatic hydrolysis with or without mechanical treatment [41]. The nanocellulose structure has a high specific surface area and good surface reactivity compared to that of macro- and micro-cellulose [42,44]. The outstanding properties of cellulose include its low density (0.0005–0.35 g/cm^3^), large specific surface area (10–975 m^2^/g), high porosity (84–99.9%), [50] high aspect ratio (length/width), high crystallinity, and excellent mechanical properties, which render it suitable for use as an adsorbent aerogel. Moreover, its limitation of disintegration upon contact with water can be addressed by combining it with a polymer such as polyvinyl alcohol (PVA) [41]. Cellulose nanofibers (CNFs) are thin cellulose fibers that have high crystallinity, great mechanical strength, and high stiffness, but a low specific surface area [24]. The limitations of CNFs include toxicity toward living things, bad environmental impact, and high energy consumption; hence, some considerations should be made when selecting CNFs [24]. A recent study has introduced a cellulose aerogel made from twisted carbon fibers of cotton via a facile route [17]. This aerogel has excellent recyclability and environment friendliness, making it suitable for industrial application. Moreover, it has an excellent absorption capacity that is superior to that of wool-based nonwoven materials, polymers, nanowire membranes, magnetic exfoliate graphite, spongy graphene, and boron-doped carbon nanotube sponges [17]. In another study, 3-D cellulose triacetate was fabricated for drug transport due to its resistance against collapse in an aqueous medium [16]. In addition, cellulose can be extracted from bacteria to afford outstanding features such as a nanofibrous 3-D porous network, high crystallinity, and superior Young’s modulus, making it suitable for use as a polymer nanocomposite [11,22].

After cellulose, the next most abundant polysaccharides are chitin and chitosan. These are built up from hydrophilic hydroxyl and amino groups, which provide excellent adsorption and surface modification capacity. Chitin can be extracted from crustaceans, usually from crab and shrimp. The structural formula of chitin is similar to that of plant cellulose, and chitosan is obtained from deacetylated chitin [51]. Chitin is popular in biomedical applications due to its biocompatibility, biodegradability, and antibacterial activity [52]. In addition to its use in drug delivery, chitin is used in catalysis, wastewater treatment, and agricultural/industrial applications [4,53]. The versatile applicability of chitin is primarily due to the presence of transformable amino groups in its molecular structure [54]. For instance, one study has shown that pristine isotropic nanochitin aerogels exhibit low densities and high porosities that are comparable to those of nanocellulose aerogels [34]. In another study, the in situ synthesis of platinum (Pt) nanoparticle on a chitin aerogel derived from doped ultra-thin carbon nanofibers (CN-Pt) was developed for application in the hydrogen evolution reaction [55]. Chitosan aerogel is superior to chitosan powder for its well-defined structure with respect to surface area, molecular weight distribution, and accessibility of the amine functional groups [56]. For the bio-functional applications of absorption and phototherapy in bacterial elimination, Zhang et al. (2020) demonstrated the embedment of chitosan aerogel with amino functionalized molybdenum disulfide nanosheets [56]. However, chitosan has some limitations such as dissolution in acids, low surface area, poor solubility, and gelation in water.

Although some reviews on aerogels have been published [57,58], none have focused specifically on the polysaccharide aerogels and bio-aerogels [24,59]. Hence, the present review is aimed at providing a comprehensive overview of the recent trends in the porous structures and synthesis of the porous polysaccharides, including a discussion of their morphological, mechanical, and thermal properties, and their current applications. In addition, the review concludes with a discussion of the future prospects and challenges in the utilization of porous polysaccharides.

## 2. Porous Polysaccharides

The physical properties of various types of polysaccharide are listed in Table 1. As much as 99% of a composite aerogel can be provided with a porous structure by processing polysaccharides into aerogel [60]. The porous structure of the aerogel provides a larger surface area for the adsorption of oils, dyes, and other compounds [13]. Moreover, the swelling behavior of an aerogel in the presence of a buffer solution indicates that the large pores enable rapid absorption and fast attainment of equilibrium, such that the swelling ratio is eventually reduced [61]. Further, the robust porous structures of the aerogel can ensure storage without subsequent leakage, and can provide good thermal insulation for maintaining the temperature at a desired level [62]. The porous structures of the aerogels provide densities that are generally below 1.0 g/cm^3^, thus leading to their classification as ultra-low density materials [3]. This occurs because their network structures contain air pockets that occupy the majority of the space within the materials. Different types of cellulose provide different pore sizes; for example, a eucalyptus aerogel has a smaller pore size compared to that of an oat aerogel [41]. The surface area of the cellulose is generally large and may contain slit pores between the fibers; however, the surface area may change during the process of crosslinking with other components.

The crosslinking of polysaccharides via physical and chemical methods can alter the physical properties of the aerogel, including the specific surface area, pore size, and density. Remarkable changes in the physical properties of the crosslinked polysaccharide aerogels have been reported via chemical methods using metal organic framework, oxalic acid or clay. For example, a novel zeolitic imidazolate framework (ZIF-67) was introduced to a bacteria cellulose/chitosan network to generate a metal organic framework, whereby the surface area was increased from 8.4 m^2^/g to 268.7 m^2^/g to afford an excellent removal efficiency of heavy metal ions and dye [22]. A similar result was obtained when chitosan was treated with oxalic acid, whereby the specific surface area was increased from the initial range of 2–30 m^2^/g to a final value of 362.30 m^2^/g. In another study, however, the addition of increasing amounts of clay to chitosan aerogel resulted in a decrease in density (<0.073 g/cm^3^) but an increase in porosity (>93%). The decrease in density was facilitated by a build-up of void spacing between the polymer molecules and the silicate layer [44]. Meanwhile, the addition of graphene oxide (GO) or silver-loaded titanium dioxide (TiO_2_) to a cellulose composite matrix has been shown to increase the thickness and decrease the pore size. This is due to the adsorption of the GO or TiO_2_ into the pores of the matrix [66,67]. Similar results were observed during the addition of the antibacterial agent, N-halamine, into the chitosan–chitin aerogel, whereby the resultant thickening of the sheets and reduction in the pore size lead to a gradual decrease in the void volume fraction within the aerogels [68]. Similarly, a decrease in the pore radius has been observed when the degree of cross-linking of the polymer chains was increased because the pore structure was created by the ionic crosslinking [69].

**Figure 3:**
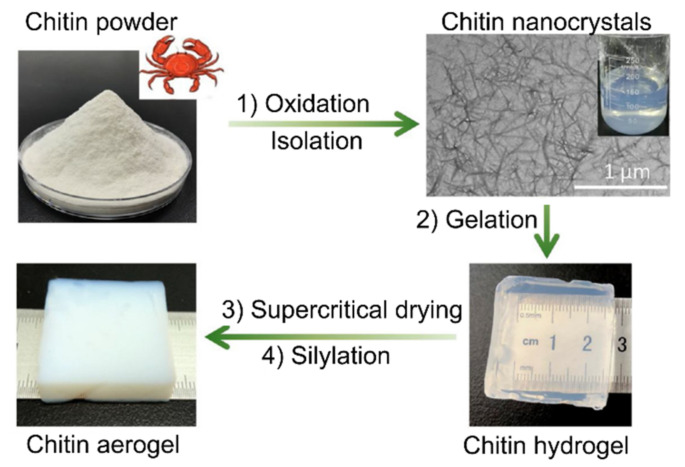
The fabrication of chitin aerogels. Reproduced with permission from reference [34]. Copyright 2020 Elsevier.

The fabrication of a chitin aerogel from chitin powder via an oxidation of the powder, isolation of chitin nanocrystals, gelation at an acidic condition, supercritical drying, and silylation process is shown in Figure 3. During the oxidation process, the chitin powder was treated using 2,2,6,6-tetramethylpiperidine-1-oxyl (TEMPO), sodium bromide (NaBr) and sodium hypochlorite (NaClO) as a catalyst to produce carboxylate groups. The pH of the solution rendered basic by introducing NaOH prior to quenching with ethanol and several cycles of centrifugation to remove all impurities. The resulting chitin nanocrystals in needle shapes with lengths of 100–400 nm and a width of 20 nm were treated with hydrochloric acid vapor for their surface carboxylates to induce gelation in acidic conditions [4].

## 3. Preparation of Porous Polysaccharides

### 3.1. Overall Preparation Process

**Figure 4:**
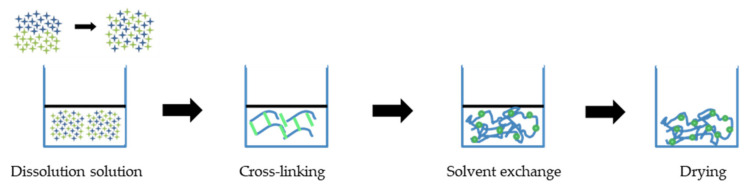
Schematic aerogel synthesis.

While many polymers are formed via hydrolysis or polycondensation, the porous olysaccharides are formed via the sol–gel process [51,59,70]. This generally involves gelation, aging, and drying as the three main steps, although the synthesis of bio-aerogels differs in that it begins with the dissolution of the polymer followed by gelation and drying, as summarized in Figure 4. The choice of aerogel preparation process depends on the nature of the initial raw polysaccharide materials. For instance, if the raw cellulose is derived from wood, the lignin must first be removed (a process known as delignification) in order to increase the porosity and chemical accessibility of the cellulose [71]. In addition, sustainable nanomaterials can be developed from cellulose via chemical and mechanical treatments. In this process, cellulose nanofibrils or nanocrystals are extracted to produce an aerogel [31]. This process begins with complete dissolution of the polysaccharide in the solvent by the application of heat for a specific time. The appropriate temperature of heating depends on the solvent; for instance, a solution in water is usually heated to 80 °C [72]. As an example, the hydrolysis of the sulphated anionic polysaccharide K-carrageenan has been achieved in 20 mL of deionized water with stirring at 85 °C in an oil-bath until complete dissolution [70]. For chitosan and bacterial cellulose (BC), a BC suspension was obtained prior to dissolution with chitosan in 1 wt.% acetic acid and stirring for 5 min [11].

Gelation is the process in which the network structure of the aerogel is formed. This can be achieved by transferring the solution into a mold and maintaining it at room temperature for 24–72 h. A study has shown that the co-gelation of multiple materials is a promising approach since it provides the aerogel with higher hydrophobicity, solubility, and biological activity [72]. The gelation of polysaccharides occurs during heating in an alkaline/urea solution, ionic liquid, or polar solvent such as ethanol or methanol. However, for the addition of metal ions to the aerogel, the metal vapor deposition method should be considered during the gelation of chitosan to allow easy re-dispersal during the transition process [65].

After the gelation step, the formed gel is immersed in the selected solvent overnight before drying. Generally, the porous structure of the aerogel is developed during the solvent removal process when drying. The solvent is generally removed at the appropriate temperature and pressures for supercritical CO_2_ drying. This is to ensure that no direct evaporation of the liquid occurs and to prevent the connected aerogel structure from shrinking. The choice of solvent can affect the physical properties of the obtained aerogel [73]; although the porosity is usually unaffected, the surface area and density have been shown to change slightly with the choice of solvent [65]. The hydrogel is formed in this step because a large amount of solvent is removed, and the coherent network structure of crosslinked polymer is synthesized. For instance, the removal of solvent from cellulose gel in a cylindrical mold has been performed in an incubator maintained at 70 °C under a heating rate of 3 °C/min for 120 min to obtain the crosslinked product [18].

**Figure 5:**
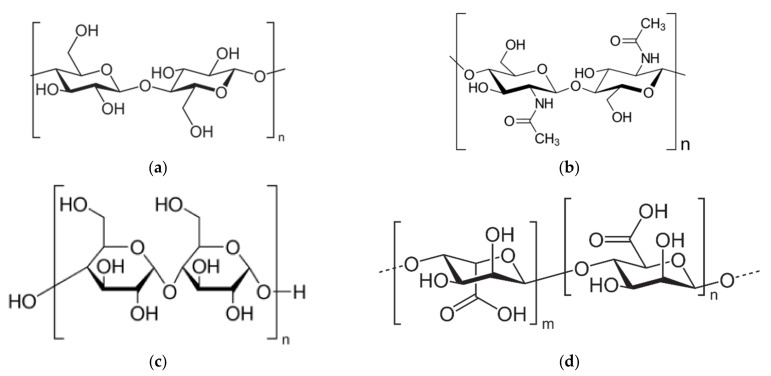
Structural formulae of (**a**) cellulose, (**b**) chitin, (**c**) starch, and (**d**) alginate.

The crosslinking process may occur via chemical (covalent bonding) or physical (ionic/hydrogen bonding) interactions between the polymer chains. This process has been shown to result in strengthening of the polymer network due to the formation of a uniform pore distribution [6]. As an example, the crosslinking of carboxymethyl cellulose (CMC) with hydroxyethyl cellulose (HEC) in the presence of divinyl sulfone (DVS) as the crosslinking agent has been studied by observing the change in color as the viscosity of the solution increased [12]. Studies have also shown that the gelation speed is faster for chemical crosslinking than for physical crosslinking, and that the chemical crosslinks generate a more stable structure [50]. Polysaccharides are capable of crosslinking or entangling with other precursors due to the presence of several functional groups on the cell wall, such as hydroxyl, phosphate, sulfate, and carboxyl groups. These functional groups are common in biomass-derived carbon, allowing it to function as good an adsorbent [55]. In addition to sustainability, these groups can provide coordination sites for various applications [2]. Further, with modifications to the coordination sites of polysaccharides, superhydrophobic properties and high absorption efficiency can be obtained. For instance, it has been demonstrated that hydrophobically modified polysaccharides can remove oils up to 60–130 times more effectively than the unmodified materials [19]. The structural formulae of various polysaccharides with –OH as a major functional group are presented in Figure 5. In the chitosan/chitin structure, –NH is an additional functional feature [64]; the deacetylation of chitosan determines the hydrophobicity, solubility, and toxicity of this group of materials.

Aerogels can be prepared directly from polysaccharide nanocrystals or polysaccharide nanocrystal composites with other polymeric matrices. The purpose of crosslinking is to improve the final mechanical properties of the aerogel [49]. For example, single cellulose is generally difficult to melt and dissolve due to its mixed crystalline and non-crystalline structure, molecular chain rigidity, and strong intra- and inter-molecular hydrogen bondings due to the –OH functional groups in the molecular chains [18]. Hence, the direct preparation of natural polysaccharide nanocrystals is difficult, except via layer-by-layer assembly [17].

**Figure 6:**
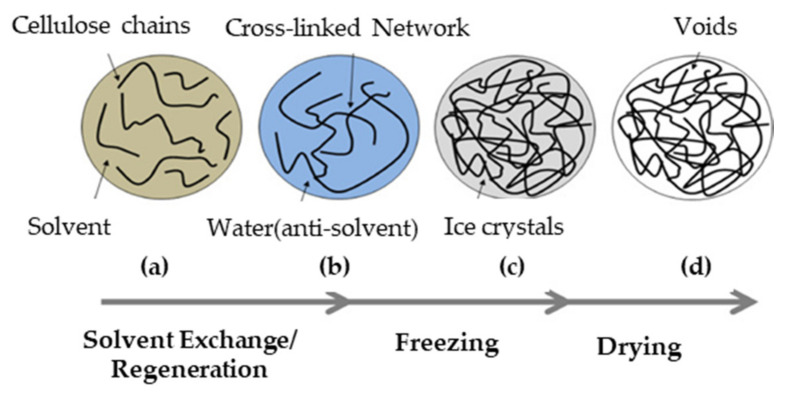
The crosslinking of cellulose to produce a polysaccharide aerogel. Reproduced from reference [77] with permission. Copyright 2012 The Royal Society of Chemistry (**a**–**d**).

The crosslinking of cellulose to produce an aerogel is shown schematically in Figure 6. During this process, a 3-D porous network structure is formed, and a solid gel is formed from the liquid. Various factors such as the type of cross-linking process (physical or chemical) must be considered along with the relevant conditions of temperature, pH, concentration, functional group, solvent, and cross-linker composition. A crosslinked aerogel polymer can be built by simple mixing, solution casting, free radical polymerization, ultraviolet (UV) and gamma ray irradiation, bulk polymerization, or interpenetration [74]. To improve the physicochemical properties of the polysaccharides, the availability of the functional groups should be increased. Significant research has been performed on the crosslinking of aerogels with other organic/inorganic materials to improve its final properties. Among the most popular inorganic materials are graphene and silica [34,60,75]. Generally, graphene is used as an elasticity material and a temperature and pressure sensor due to its high electrical conductivity. A recent study showed that the addition of graphene oxide (GO) into collagen provides good biocompatibility and osteogenic ability in vivo, which is promising for medical applications such as bone regeneration and tissue engineering [34]. Similarly, silver nanoparticles have important antimicrobial properties for medical and packaging purposes [49,65]. With the recent development of a novel aerogel from a quaternized *N*-halamine silane monomer, nanocrystalline cellulose, and cellulose, it has become possible to achieve a high efficiency of over 99.9% for oil/water separation [68]. It has also been reported that a cellulose aerogel with molybdenum disulfide can provide enhanced adsorption of particulate matter [76]. In another study, the covalent crosslinking of polyamide and epichlorohydrin on carbon nanofiber (CNF) has been performed to generate a stable 3-D structured cellulose network [63].

The chemical modification of cellulose can be performed via the processes of etherification, esterification, oxidation, or grafting with various ligands. For example, cellulose has been modified by dissolving in NaOH/urea/water, and crosslinking with *N*,*N*′-methylene bisacrylamide or graphene oxide [67,78,79]. For antimicrobial cellulose, particularly for use in fibers and fabrics, copper (Cu), zinc (Zn), or noble metals such as silver (Ag), gold (Au), and platinum (Pt) are incorporated [60,80]. For use in CO_2_ absorption applications, amine is grafted onto the cellulose network structure via the –OH groups [15,63]. Meanwhile, the crosslinking of chitosan aerogel with polyvinyl alcohol (PVA) has been shown to inhibit aerogel shrinkage by 20% due to supramolecular effect of PVA [81]. Chemicals such as aldehydes, genipin, formaldehyde, glyoxal, and glutaraldehyde have been used in the chemical crosslinking of chitosan [64]. It should be noted, however, that a recent study comparing the sol–gel/crosslinking method with the alternative hydrothermal method showed that the latter could be used to produce a chitosan-graphene aerogel with a higher methyl-orange absorption rate of 96.6% [23]. In addition, a low temperature of 140 °C during hydrothermal treatment has been shown to result in a higher removal efficiency of metal ions compared to that obtained at the higher temperatures of 180 and 215 °C due to an increase in the crystallinity of the sample obtained at low temperature [20].

The physical gelation process can be performed for chitosan via hydrogen bonding, which can be influenced by the pH of the solution. For example, before drying, the chitosan can be dissolved in a bath containing a multivalent acid (e.g., oxalic acid), or a solution with high ionic strength can be used due to its low cost and wide applicability as a reagent in the biomedical field. Interesting results have been obtained by using chitosan with a higher porosity and a high water uptake. In addition, some studies have combined chemical and physical (ionic) crosslinking to provide intramolecular hydrogen bonding and, thus, improve the swelling behavior [60]. Meanwhile, Ko and Kim (2020) compared the chemical crosslinking of chitosan and epichlorohydrin with the ionic crosslinking in the presence of itaconic acid to demonstrate advantages of the ionic liquid such as a negligible vapor pressure, high thermal and chemical stability, good recyclability, high electrical conductivity, and good solubility [61,68,82]. Other modifications of chitin/chitosan include the substitution of silver nanoparticles [65], vancomycin [83], metal organic frameworks [22], nano silicon/particles [11,66,84], and polybenzoxazine/clay [44]. For applications in the adsorption of pollutants, chitin/chitosan have been modified with glutaraldehyde, formaldehyde, tripolyphosphate, polyaspartic acid sodium salt, and persulfate [85].

The various crosslinking processes of polysaccharides are listed, along with the corresponding organic/inorganic reagents and drying methods, in Table 2. Although UV irradiation is also a possible crosslinking method for aerogels, no specific study has been performed on the application of UV to the polysaccharide aerogel. The composition of the polymeric substance (i.e., the type of polysaccharide) will influence the final properties of the aerogel, including the surface area, pore volume, and pore diameter.

The final step in the preparation of porous polysaccharide aerogels is the drying process whereby the hydrogel is converted to an aerogel, the surface tension is minimized, and the porous structure is developed. This is the most critical step since the pores of the hydrogel are filled with liquid (solvent) which must be removed without damaging the microstructure. During this stage, the pore walls experience increased capillary tension at the interface between the liquid and solid surface. The morphology, porosity, and structural integrity of the final structure depend entirely on this step. Thus, a suitable drying method such as freeze drying or supercritical carbon dioxide, or rarely used methods such as ambient pressure drying, vacuum drying, and microwave drying, should be employed [86]. For example, the process of starch-based aerogel formation begins with swelling when the starch granules absorb water, followed by gelatinization as the amylose molecules are leached, then destruction of the granular structure by heating, followed by retrogradation, restriction, partial recrystallization, and degradation [6]. Studies have shown that a transparent hydrogel can be obtained by increasing the concentration of carbohydrates (galactose, glucose, sucrose, and trehalose) [45]. Moreover, the hydrogel transparency can be controlled by the heating temperature and the composition of the coagulation bath during the gelation process. The gel is immersed in the initial drying solvent under specific conditions of time, density, and temperature change. Finally, the aging process can enhance the mechanical strength of the tenuous aerogel network. This is important for producing a compact structure that is resistant to damage and shrinkage [11,84].

To shape the aerogel into a desired structure, e.g., droplets or fibers, an additional step is required. The formation of droplet aerogels can be achieved by a conventional dropping method using pipettes and syringes, an emulsification method, or a thermal injection method [87]. Wet or spinning processes are generally used for the synthesis of aerogel graphene-chitosan fibers [88]. To meet industrial demands, revised methods such as the vibrating nozzle method and the electrostatic and mechanical cutting methods should be used [51,89]. Two approaches have been used in the past for porous aerogel fabrication, namely: supercritical CO_2_ (sc-CO_2_) drying and freeze drying (FD). Each of these processes has advantages and disadvantages, which are described and compared in the following sections.

### 3.2. Drying Technologies

One of the most challenging aspects of aerogel production is the extraction of the liquid solvent in order to avoid the degradation of the nano porous structure, which would otherwise lead to cracking and shrinkage of the dried gel. Hence, the selection of the best drying method is critical to the development of the aerogel. In the present review, the following drying technologies were identified as the most commonly used.

The supercritical CO_2_ (sc-CO_2_) drying process uses CO_2_ to transform the organic solvent in the polymeric hydrogel into a supercritical solution, thus allowing the gel to form an aerogel. This drying process provides a well-defined structure by preventing the shrinkage or collapse of the mesopores by achieving zero surface tension (i.e., supercriticality) [12]. In addition, the rheological behavior of the aerogels can be controlled by modifying the experimental conditions during the sc-CO_2_ drying process [96]. Aerogel formation via sc-CO_2_ involves three steps, namely: gelatinization, alcogel formation, and drying [14]. Solvent exchange occurs at the alcogel attainment step, as organic solvents such as ethanol and water are generally used in different concentration ratios [12,14]. For example, Baldino et al. studied the effect of the ethanol: water ratio to demonstrate that the nanofibrous morphology of the native CMC gel can be preserved via solvent exchange in 50% *v*/*v* ethanol/water. Further, the swelling ratio was reported to be 20% higher than that of previous studies, and the water uptake was 500 times larger under the same conditions [12]. The important conditions that must be controlled in the last stages are the critical temperature and pressure. By using CO_2_ as the fluid, the polysaccharide gels could be dried under the mild critical point conditions of 304 K and 7.4 MPa. Since the polysaccharide gels are temperature-dependent, the low temperature used for sc-CO_2_ (310−330 K) could decrease the changes occurring at the molecular level (e.g., hydrogen bonding between the molecules). The sc-CO_2_ is transferred to the liquid gel solvent, thus causing expansion, and the excess liquid volume is then removed from the gel network. By this process, the amount of CO_2_ in the pore gel liquid is gradually increased until supercritical conditions are achieved without any vapor-liquid transition [97]. Finally, slow depressurization to atmospheric pressure must be performed so that the aerogel can be recovered. In one study, for example, the remaining ethanol was removed from a starch aerogel by setting the autoclave to a temperature of 37 °C and a pressure that was 80 bar above the critical point with a CO_2_ flow rate of 5 kg/h for 5 h. Depressurization was then performed at a slow rate of 5 bar/h to prevent shrinkage of the sample before final cooling to room temperature [98]. The sc-CO_2_ drying is performed under high temperature and pressure, consumes a large amount of solvent, and requires a significant amount of time for solvent exchange, thus increasing the overall cost [1]. For example, cellulose aerogel has been dried using CO_2_ for 3.5 h in an autoclave at 40 °C and 120 bar [11,83], and chitosan aerogel has been dried for 4 h under the same conditions [25]. To study the effect of sc-CO_2_ drying upon a bi-component natural aerogel, Baldino et al. introduced alginate-gelatin and chitosan-gelation. The results demonstrated that uniform gels were produced with the preservation of a delicate structural morphology. This is attributed to the near-zero surface tension of the supercritical fluid mixture of CO_2_ and organic solvent during the sc-CO_2_ drying process. The results also demonstrated that a high porosity of more than 80% could be achieved [99].

**Figure 7:**
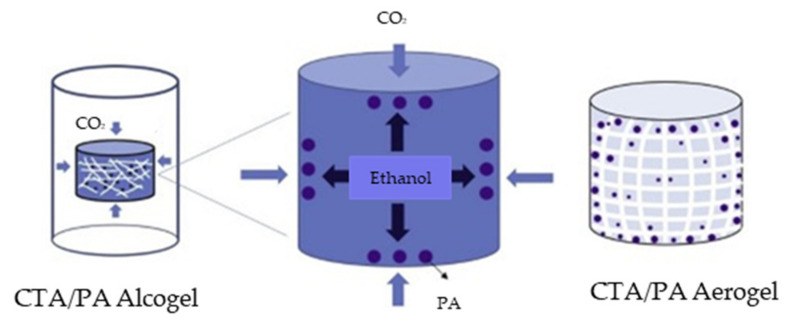
A schematic diagram of the preparation and sc-CO_2_ drying of a cellulose triacetate/paracetamol CTA/PA aerogel. Reproduced from reference [100] with permission. Copyright 2020 Elsevier.

The formation and sc-CO_2_ drying of a cellulose triacetate (CTA)/paracetamol(PA) aerogel is shown schematically in Figure 7. First, the CTA/PA aerogel was dissolved in ethanol to produce a homogeneous mixture and allow the polymer gel to form. The polymer gel was then dried under sc-CO_2_ at 15 MPa and 40 °C without changing the solvent. During the drying process, the aerogel was observed to shrink due to the low capillary force between the CTA and solvent.

The process of freeze drying (FD) can be divided into the two stages of pre-freezing and lyphilization. In the pre-freezing step, the liquid in the wet gel is either quickly frozen using liquid at −196 °C for 10 s or slowly frozen over a period of 24 h using a freezer at −18 °C. This is followed by the lyphilization step, in which desorption occurs and the moisture content is reduced from 7% to between 0.5 and 2.0%. In this step, the frozen gel is dried via sublimation at −45 °C and 15 Pa for 48 h as the gas replaces the liquid in the gel, thus generating the porous structure under vacuum. The FD method depends on the solvent crystallization temperature at the freezing point. The freezing point of the solvent should be higher in the solid–liquid phase than in liquid-liquid phase. As the temperature of the polymer is lowered, the solvent crystallizes, and the polymer separates out. The crystallized solvent is then separated during sublimation, thus generating pores with a similar morphology to that of the solvent crystallites. Therefore, the pore size can be controlled by controlling the freezing point of the solvent. The FD process is performed for two reasons: (i) as a stabilizing technique for the gel body, and (ii) to facilitate solvent exchange in order to provide a high sublimation pressure and a low coefficient of expansion. In addition, the porosity of the aerogel and its orientation depend on the direction of ice crystal growth, the freezing rate, and the freezing temperature [63]. When the freezing temperature decreases during the drying process, the pore size is increased. In one study, for example, the freezing temperature was varied between −10 to −70 °C to find that the pore size decreased with increasing temperature up to −50 °C, after which no significant change of pore size was observed [48]. After the freezing process, the solvent generally forms a dry powder, which is removed by vacuum or hydrothermal treatment. In addition, the effects of the temperature, time, and direction of ice growth during freezing will affect the physical and mechanical properties of the aerogel [101]. For instance, pore structures with different diameters are formed under high- or low-pressure freeze drying. A high freeze drying pressure generates a scaffold with a small mean pore diameter of 55 ± 4 µm and a porosity of 33 ± 12%, whereas a low freeze drying pressure generates a scaffold with a large mean pore diameter of 243 ± 14 µm and a high porosity of 68 ± 3% [96]. Another study demonstrated that the porosity of the aerogel scaffold can be controlled by combining chemically crosslinked polysaccharides with the non-toxic cyclic triphosphate sodium trimetaphosphate (STMP) under alkaline conditions followed by physical crosslinking via the FD process [102].

#### Comparison of sc-CO_2_ and Freeze Drying

**Figure 8:**
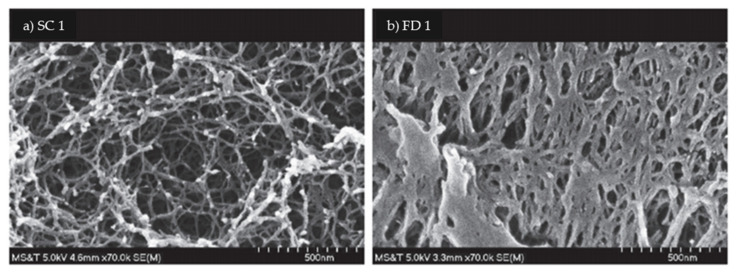
Scanning electron microscope (SEM) images comparing the structures of polyurea aerogels produced via (**a**) sc-CO_2_ drying and (**b**) freeze drying (FD). Reproduced from reference [103] with permission. Copyright 2018 Springer.

Both the sc-CO_2_ and freeze drying (FD) methods are widely used to produce aerogels; however, each process has its own advantages and disadvantages, such that the method of choice depends on the specific requirements and intended application of the aerogel. To illustrate this, the structures of polyurea obtained via sc-CO_2_ drying and FD are compared by the scanning electron microscopy (SEM) images in Figure 8 [103]. Here, the sc-CO_2_ drying is seen to provide a homogeneous fibrillar structure, whereas the FD results in a denser structure with high fiber aggregation caused by the growth of large solvent crystals during freezing. This is because the liquid solvent diffuses from the smaller pores to the larger ones during the FD process, thus causing tension in the walls of the larger pores and generating a denser fiber network. Conversely, the sc-CO_2_ drying process provides a finer pore size and a narrower pore-size distribution due to the absence of any apparent or occasional aggregation of the fibers at low concentration and to the absence of surface tension which prevents the collapse of the pores during solvent elimination [97]. Thus, the sc-CO_2_ drying provides a well-defined structure by preventing shrinkage or collapse of the mesopores during drying. However, the sc-CO_2_ drying is performed under high temperature and pressure, consumes a large amount of solvent, and requires a significant amount of time for the exchange of solvents, thus increasing the overall cost [1]. Thus, the sc-CO_2_ process is more expensive than the FD process [103]. These factors also make it difficult to scale up the sc-CO_2_ drying process to either the pilot or industrial scale. Further, the sc-CO_2_ drying process produces aerogels with smaller macropores than those obtained via FD. In terms of the mechanical properties, the compressive strength of the CNF aerogel product obtained via freeze drying is greater than that obtained via sc-CO_2_ drying due to the formation of the ice crystals that enhance the internal structure of the aerogel via cellulose aggregation [104]. Moreover, the FD method is more favorable due to its ease of application, environmental friendliness, and cost effectiveness. The aerogel from FD shows a higher shrinkage, higher pore volume, low specific surface area, and low density. For instance, Czlonka et al. (2018) demonstrated that the FD aerogels shrank by 10–12% whereas the sc-CO_2_ aerogels shrank by only 8–9% [103]. In another study, the aerogel obtained via sc-CO_2_ was shown to have a lower density (0.009–0.05 g/cm^3^) and higher surface area (72–115 m^2^/g) compared to that obtained via the FD process [105]. Nevertheless, the FD process is also time consuming due to the long aging period of more than 2 days. Moreover, the FD process causes the destruction of the network structure during the crystallization of the pore liquids [106]. To resolve this issue during the fabrication of cellulose aerogels, a study was conducted to address the limitations of the process under ambient pressure by introducing naphthalene crystals to promote pore formation [106]. Nevertheless, both of these traditional techniques suffer from the lack of reproducible customization of the 3-D aerogel structures and the lack of easy fabrication of complex structures. One possible solution to these limitations is to combine the process of freeze drying with that of cross-linking. This novel approach can offer additional advantages, as reported by Autissier et al. (2010). In his study, a polysaccharide gel solution was freeze dried in the presence of a 30% *w*/*v* sodium trimetaphosphate cross-linking agent and the drying pressure was adjusted to facilitate cross-linking of the polysaccharides and, thus, induce porosity without the need of a solvent. Thus, the use of organic solvent was reduced while controlling the porosity and architectural structure of aerogel [102]. A comparison of the effects of the hydrogel concentration (1–2 wt.%) upon the formation of a lupin hull cellulose nanofiber aerogel has been performed using both the scCO_2_ and FD processes. The results indicated that the lowest density (0.009–0.05 g/cm^3^) was obtained for the aerogel made from the lowest initial hydrogel concentration of 1 wt.%, but the surface area was relatively high (72–115 m^2^/g) [105].

## 4. Characterization of the Polysaccharide Aerogels

### 4.1. Morphological Studies

The network structure and shapes of the pores in the aerogel can be measured using scanning electron microscopy (SEM). The characteristics of pore size and distribution are dependent on various factors such as the drying process and the concentrations of the aerogel component. The study of aerogel morphology examines the important aspect of the network structure that links the constituents to each other and, thus, forms the porous structure [25]. In addition, the drying efficiency during aerogel formation can be observed by examining the developing morphology over several works. Such studies clearly indicate that the structure can be destroyed during the drying-adsorption cycle, particularly when the FD methods are used, due to the rapid change of liquid to crystal form [104]. In contrast, the sc-CO_2_ drying can maintain the original pore size of the aerogel [25,103]. During the sc-CO_2_ drying, the gas–liquid interface and surface tension disappear and, hence, the destruction of the internal 3-D structure is avoided. The effects of gelatination due to the entanglement of biopolymer fibrils during aerogel formation also can be observed via SEM. The entangled fibrils generate a multi-level supramolecular organization in which the small particles are located inside the fibrils.

The inclusion of additives in the aerogel structure can also cause variations in the network structures. For instance, Jaafar et al. observed that the addition of xyloglucan (XG) in a cellulose nanocrystal (CNC) aerogel altered the morphology from a lamellar to an alveolar form due to an increase in the viscosity of the XG/CNC colloidal dispersion [107]. In another study, an increase in the CNF concentration from 2.5 to 3.5 wt.% was shown to create a relatively dense aerogel [104]. The same result was observed for a chitosan aerogel, where a dense, regular, and consistent pore structure was obtained when amino-functionalized molybdenum disulfide was embedded [57] and when the concentration of chitosan was high [25]. This densification occurs because the filler occupies part of the pore network. In another study, a cellulose aerogel was impregnated with a silica precursor, followed by in situ formation of the silica gel additive, thus resulting in an interpenetrated cellulose-silica network [45].

### 4.2. Mechanical Properties

**Figure 9:**
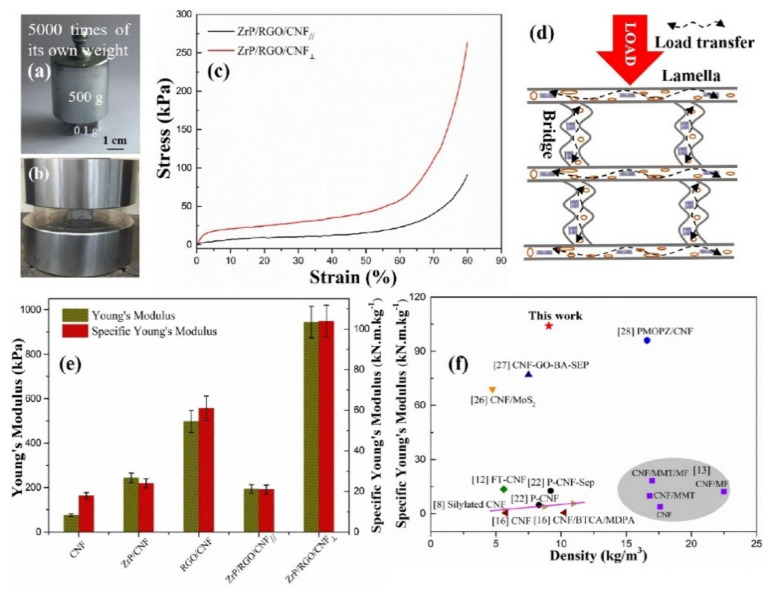
Mechanical properties of biomimetic–structural ZrP/RGO/CNF aerogel. (**a**) Optical image of an aerogel sample. (**b**) Photograph of a cubic sample in the compressive test. (**c**) Stress–strain curves of aerogels under different compression loads, perpendicular and parallel to lamellar alignments. (**d**) Schematic illustration of the load transfer perpendicular to lamella alignments throughout aerogel. (**e**) Young’s modulus of aerogels. (**f**) The comparison of specific Young’s moduli of ZrP/RGO/CNF aerogel with other cellulose nanofiber-based aerogels from previous work. Reproduced from reference [112] with permission. Copyright 2020 Elsevier.

The mechanical characterization of aerogels is often difficult, either in terms of specimen preparation or accurately measuring the small forces of applied load. Thus, texture analyzers are commonly used in compression and flexural modes for testing the mechanical properties of polysaccharide aerogels. As a representative method, the compressive testing is used to investigate the mechanical properties of aerogel, yielding results such as recovery rate and stress–strain curves. The aerogel samples were typically prepared using a cylindrical [108] or cubical mold [109] in which hydrogels are placed and dried. The majority of polysaccharide aerogels were tested in the uniaxial compression mode with the cylindrical samples [109,110]. Generally, the compression mode is set under a small load (0.1–30 kN) with a discoidal probe, compression rate (2–3 mm/min) and ratio height/diameter ratio of 1.5:1 [109,110,111]. Figure 9 shows that the anisotropic architecture of ZrP/RGO/CNF aerogel is responsible for the anisotropic compression action of under various load orientations parallel and perpendicular to lamellar alignments [112].

Polysaccharide aerogels with a low density and a high porosity are inherently fragile and brittle, resulting in poor mechanical properties. Thus, enhancing the mechanical properties of the polysaccharides via control of their microstructures is important for their potential applications. Any addition of a foreign material to the polysaccharide aerogel matrix is expected to increase the ratio of the compressive strength to the compressive strain [78]. A cellulose-based aerogel does not readily return to its original state after compression because the pressure causes some damage to the structure. However, when chitosan is added, the aerogel exhibits a more flexible and resilient structure as the chitosan support adds strength and structural integrity to the cellulose skeleton [57,110]. Studies have shown that the uneven distribution and irregularity of the pores contribute to the poor mechanical properties of starch aerogels, whereas the addition of non-starch ingredients such as konjac glucomannan, sodium palmitate complexes, poly-(3,4-ethylenedioxythiophene) and k-carrageenan, provide enhanced mechanical properties [6]. The resulting increase in the Young’s modulus and yield stress might be due to an increase in the thickness and elasticity of the cell wall, along with a narrowing of the pore-size distribution. Similar results were obtained by Qin et al. (2020) when the addition of a reduced graphene oxide (r-GO) support on carbon fiber (CF) enhanced the mechanical compressive properties of the aerogel via covalent crosslinking of the r-GO to create a 3-D network structure. A study shows that at maximum value, the compressive stress is 55.8 kPa and compressive strain is 66% for *N*-doped CF/r-GO [93]. Similarly when cellulose nanofibers were added to the aerogel, its compressive stress, Young’s modulus, and yield stress were increased. However, the addition of nano-silicon to a chitosan aerogel resulted in a decrease in flexibility and tensile strength due to the strong interactions between the chitosan chain and the small SiO_2_ units, which eventually altered the compatibility with the biopolymer matrix [66]. Further, owing to the abundance of –OH groups in starch, the mechanical strength is affected by moisture absorbance (van der Waals interaction), which results in a loss of functionality [6,108]. Comparisons between the compressive stress–strain curves of organic and synthetic aerogels have revealed that the aerogel from 1 wt.% hydroxyl ethyl cellulose (HEC) is comparable to the alumina aerogel. However, the elastic modulus was found to dramatically increase from 0.027 to 0.553 MPa with increasing HEC content [111].

### 4.3. Antibacterial Properties

The study of the properties of polysaccharide aerogels is important for preventing bacterial growth in treated wounds and for providing a clean environment for biomedical applications. The antimicrobial properties of polysaccharide aerogels have been investigated by cutting the aerogel into a disk on agar medium followed by the addition of bacteria. The majority of studies on antimicrobial properties have generally employed inorganic metal ions such as silver (Ag) and zinc oxide (ZnO), or silicon dioxide (SiO_2_) nanoparticles on the surface of the polysaccharides for increased inhibition. The addition of SiO_2_ nanoparticles to a chitosan aerogel was shown to provide the maximum inhibition of *E. coli* and *S. aureus* by binding to, and disturbing the permeability of, the bacterial cell wall [60]. In addition, it can pass through the bacterial cell wall and interact with the DNA and proteins to cause further damage. For example, Ag ions react with the positively and negatively charged sites on the outer cell membrane, thus causing changes and damage to its surface [95]. Studies have also found that the extent of the inhibition of *E. coli* and *S. auereus* by ZnO nanoparticles (as indicated by the inhibition diameter on the agar gel) varies with the concentration of the ZnO. Thus, the inhibition diameter was observed to increase with increasing concentration of ZnO, and was higher for *E. coli* than for *S. auereus*. This may be due to a reduced penetration of the nanoparticles by the thicker and more complex peptidoglycan structure of *S. auereus* as opposed to an enhanced transportation of the nanoparticles by the negatively charged *E. coli* [40]. The antibacterial effect can be also improved by the addition of organic species. For instance, while pure nanocellulose has no effect on *E. coli* and *S. aureus*, the addition of chitosan leads to damage to the bacterial cell walls via interactions between the positively charged chitosan and the negatively charged microbial cell membranes or by penetration of the antibacterial agent into the cell nuclei and inhibition of protein synthesis [66].

### 4.4. Thermal Properties

The thermal conductivity of the polysaccharide aerogel is dependent on the type of polysaccharide and the processing conditions, which also influence the pore size. The pore size significantly influences the mechanism of heat transfer via thermal conduction. Aerogels contain a gas phase that decreases the amount of collisions between the molecules, thus decreasing the thermal conductivity and limiting convective mass transfer [31,36]. The thermal conductivity decreases with an increase in the density and a decrease in the porosity. In particular, the low thermal conductivity of starch has been related to the observation that the pore size is less than the mean free path of air molecules [6]. Furthermore, the thermal properties of the aerogel change depending on the presence and types of additive. For instance, while the thermal conductivity of a pure cellulose aerogel was found to be 0.033 W/m K, the addition of silica reduced this value to 0.027 W/m K [62]. This was attributed to the inhibition of heat conduction in both the solid and gas phases due to the amorphous slender skeleton of the silica aerogel. Similarly, the thermal conductivity of a cellulose nanofiber (CNF) aerogel was reduced from of 49 mW/m K to 18 mW/m K with the addition of zirconium phosphate/reduced graphene oxide (ZrP/r-GO/CNF) as the mesoporous structure of the additive provided more free space [104]. It has also been demonstrated that the thermal stability of CNF is improved by the addition of ZrP/r-GO, thus increasing the decomposition temperature, decreasing the decomposition rate, and producing more char residue [109]. Studies have revealed that the change in thermal stability is due to the modification of the methyl group, which affects the bond length and structure [108]. It has been observed that the thermal stability of bacterial cellulose (BC)/GO can be enhanced compared to the original BC due to the presence of a strong crosslinked network generated by the interactions between the GO and BC [11]. In another study, the decomposition rate and weight loss were decreased when titanium dioxide (TiO_2_) was grafted onto the CNF aerogel due to an increase in the level of cross-linking and the formation of a compact structure [112].

### 4.5. Wetting and Solubility

The study of aerogel wetting and solubility is important for the absorption of oil and the transportation of chemicals (e.g., drugs) in biomedical applications. The wetting behavior or hydrophobic stability of polysaccharides for oil separation can be evaluated by measuring the change in contact angle over time. The stability of a water droplet on the material surface can then be calculated using the Gibbs free energy, ΔG. An increase in ΔG generates a high contact angle and prevents the water droplet from spreading on the surface of the aerogel [63]. By contrast, an aerogel intended for filtration applications must exhibit the superwetting properties in order to break down the emulsion. Thus, it is essential to develop an understanding and characterization of the aerogel surface properties as hydrophobic–oleophilic, hydrophilic–oleophobic, or omniphobic [113]. Hydrophobic and lipophilic properties provide the aerogel with good absorption capacity, and super-hydrophobicity that can be achieved via modification with silane coupling agents. Thus, in one study, the super-hydrophobicity of a silane-coated cellulose aerogel was demonstrated by an average contact angle of 151 ± 7° over a measurement period of 300 s [63]. However, the performance of an oleophilic and superhydrophobic aerogel may become compromised due to oil contamination and a resultant blockage of the pores during separation and circulation. The adherence of oil to the aerogel surface arises due to the intramolecular interactions and van der Waals forces. For instance, Zhang et al. reported that the addition of *N*-halamine to a cellulose–chitosan aerogel increased the aerogel density and decreased its porosity to generate highly oleophobic properties [68].

## 5. Applications

Aerogels can be synthesized from various polysaccharide sources depending on the intended application. As shown in Table 3, cellulose is the most favorable polysaccharide for a range of applications due to its unique properties. The wide application of aerogels, including drug delivery, tissue regeneration, chemical adsorption, thermal insulating materials, wound dressings, and air filtration, is the main reason of the extensive study in this area. Each of these applications is discussed in the following paragraphs.

**Figure 10:**
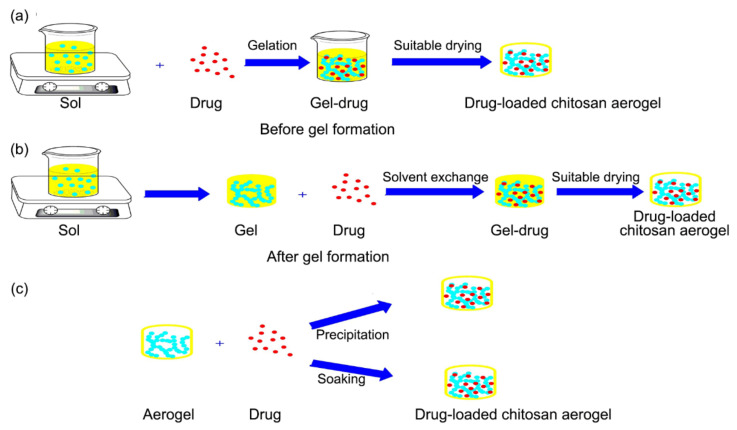
Drug-loaded chitosan aerogel: drug adding (**a**) before gel formation, (**b**) after gel formation, and (**c**) the addition of drug during adsorption/precipitation in the as-prepared chitosan aerogels. Reproduced from reference [64] with permission. Copyright © 2020 Elsevier.

In drug delivery, the nanostructured solid aerogel provides a large pore volume and a favorable surface area (larger inner surface area and high surface-to-volume ratio), making the loading of active compounds such as pyrimidine possible [118]. Furthermore, the outstanding characteristics of polysaccharides, including their biocompatibility and pH-responsive behavior, make them good candidates for the drug delivery application [119]. In addition, the pore size of the polysaccharide aerogel can be adjusted for drug storage [120] while sufficient resistance to shrinkage and collapse (depending on the drying process) allows it to maintain the pore structure [121]. Cellulose is one of the most popular polysaccharides for biomedical applications because it is the most abundant biopolymer in nature and is biocompatible, biodegradable, and non-toxic. Recently, nanocrystalline cellulose (NCC) has been applied in diagnosis, sensing, tissue engineering, and drug delivery. Both hydrophobic and hydrophilic drugs can be loaded onto the NCC surfaces by suitable surface modification with cetrimonium bromide or polyethylene glycol. In addition, a large quantity of drugs or active agents can be loaded into electro-spun cellulose nanofibers with a core–shell structure, thus increasing the stability of the drug [122]. Furthermore, the hygroscopic and swelling characteristics of this material lead to efficient drug loading [123]. Meanwhile, the biocompatibility, biodegradability, and non-toxicity of the chitosan aerogel make it a promising candidate for a drug delivery system in living organisms [64]. Chitosan can affect fat metabolism in the body and ameliorate the aqueous solubility of poorly soluble drugs. In drug delivery, the pH-responsive features of the aerogel makes it effective for releasing the drug into acidic tumor tissues. As shown schematically in Figure 10, the drug can be loaded into the aerogel either during the sol-gel process or after drying of the aerogel matrix. Moreover, loading during the sol-gel process can be performed either (i) before gelation/solvent exchange, where the active compound can be loaded into the sol prior to gel formation (co-gelation), or (ii) during the solvent exchange process via the dissolution and adsorption of the active compound from the fresh solvent in the wet gel structure. Of these, method (i) is considered to be the simplest and most flexible, while method (ii) requires an additional post-processing step in order to load the target compound into the aerogel host matrix. In addition, the selection of the appropriate medium for the drug loading stage is an important processing parameter to consider [97]. The loaded material is then dried under vacuum until a constant weight is achieved. The advantages of this method are its simplicity and flexibility, but it has the limitation of a possible reaction between the drug molecules and the chemicals used to form the gels. Alternatively, the drug molecules can be loaded after gel formation by soaking the drug molecules in the gel. The drug molecules diffuse into the gel pores and the material is then dried under sc-CO_2_ conditions. This efficient method can save time and, thus, reduce the cost of fabrication. However, the diffusion rate depends on the size of the pores and the drug molecules. Also, the initial aging solution should be further improved as it may affect the processing time [124].

Polysaccharides such as chitin and chitosan can be used for tendon tissue regeneration because as hydrophilic polymers, they can support cell adhesion and proliferation, as reviewed in the literature [125]. Biological scaffolds that resembled natural extracellular matrix were prepared based on polysaccharides via an electrospinning or freeze drying process. However, these processes suffer from missing hierarchical, porous structures, and poor mechanical properties. The supercritical gel drying process that enabled one to form different scaffold substructures with a gradient of cell populations was suggested for mimicking nanofibrous structures of natural tendon scaffolds.

**Figure 11:**
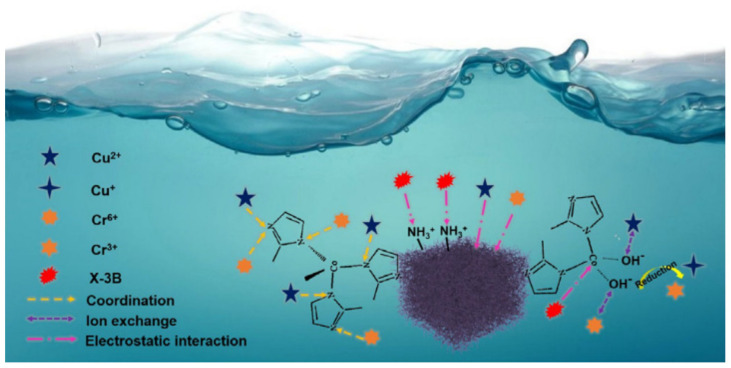
The bacterial cellulose/chitosan (BC/CH) composite aerogel modified with the zeolitic imidazolate framewok-67 (ZIF-67) for the removal of heavy metal ions and organic dyes. Reproduced from reference [22] with permission. Copyright 2019 Elsevier.

The aerogel is used in the adsorption process due to its high porosity, large surface area, and adjustable framework. The process of adsorption for the removal of contaminants such as dyes or heavy metal ions from wastewater can be achieved because of the availability of pore space, and the adsorption capacity can be enhanced by grafting and incorporating solid adsorbents [85]. The functional groups in the polysaccharide structure itself can also provide functionalities for adsorption. For instance, chitosan has abundant amine (NH) and hydroxyl (OH) groups that contribute space for ion exchange with heavy metal ions and anionic organic pollutants [126]. The recyclability of polysaccharide aerogels could be improved by developing magnetic carboxymethyl chitosan (Fe_3_O_4_@chitosan) via a mussel-inspired polydopamine chemistry with a glutaraldehyde crosslinker [127]. In another study, a graphene oxide (GO)/aminated lignin aerogel was able to remove 91.72% of dyes. The improvement of the absorption efficiency was attributed to the cross-linking between the lignin and GO, which also provides a supporting skeleton and increases the specific surface area [128]. In addition, the hydrophobic modification of the cellulose fiber provides a low surface energy, thus generating a porous sponge that can adsorb more organic solvents and oils from polluted water [19,21,63]. For sustainable design, an important criterion of a good adsorbent is its reusability. In this respect, cellulose aerogel has been shown to be reusable for up to 10 times after the adsorption of oil, with a 12.55% decrease in efficiency [3]. In adsorption experiments, the effect of pH, contact time, initial concentration, adsorbent dosage and ionic strength should also be considered. For example, a multifunctional composite aerogel adsorbent has been developed from zeolite imidazolate framework-67 (ZIF-67) by the modification of the metal–organic framework (MOF) with bacterial cellulose/chitosan (BC/CH) using the facile methods of physical mixing, in-situ synthesis, and lyophilization [24]. It was reported that the adsorption capacities of the composite aerogels for Cu^2+^ and Cr^6+^ were 200.6 mg/g and 152.1 mg/g, respectively. In addition, the good potential of the material for use in wastewater treatment was demonstrated by a 100% removal of the active dye, red X-38. This is because the incorporation of MOF increased the porosity of the BC/CH aerogel, thus providing additional space for adsorption [22]. Figure 11 shows the removal process of various metal ions using the three distinct methods of coordination, ion exchange, and electrostatic interactions with the ZIF-67/BC/CH aerogel. Thus, the Cu^2+^ and Cr^6+^ can become attached to the aerogel via both coordination and ion exchange, while the adsorption of X-38 occurs through the electrostatic interaction.

Aerogels such as cellulose-based composite sponges are excellent thermal insulating materials for use in energy efficient buildings due to the low thermal conductivity of air in the pore spaces and the negligible convective and radiative heat transfer through the gas phase [129]. However, some limitations on the practicality of aerogel as a thermal insulator should be considered, including its mechanical brittleness, high energy requirements, and time-consuming fabrication process [130]. A recent example of an aerogel that combines thermal insulation with high mechanical strength is the nanofibrous Kevlar (KNF) aerogel in the form of threads. This newly developed aerogel has excellent thermal insulation under extreme conditions (−196 or +300 °C) due to its three dimensional interconnected porous network structure [33]. To maintain the mechanical performance of the aerogel while achieving low thermal conductivity, polyaniline, graphene, silica, and clay have also been combined with the polysaccharides [34,110]. The thermal conductivities of various types of porous materials have been compared. The thermal conductivity of CNF xerogel is within the suitable range for flexible polymer foam and higher than that of silica and CNF aerogels and of the rigid polymer foams reported in other studies [131].

**Figure 12:**
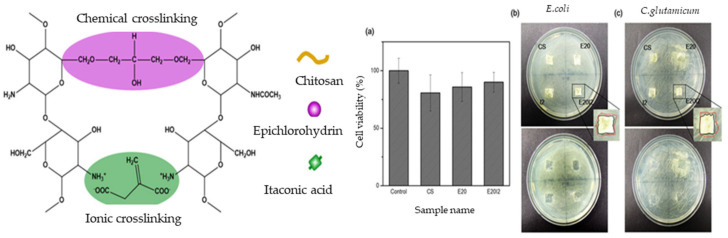
Schematic representation of chitosan/epichlorohydrin/itaconic acid for wound dressing (testing on cell viability and antibacterial activities. (**a**) cell viability of the aerogels against HT29 by MTT assay, and inhibition zones of the aerogels against (**b**) *E. coli*, (**c**) *C. glutamicum* for actibacterial activities. Reproduced from reference [61] with permission. Copyright 2020 Elsevier.

Wounds or skin injuries are complex and, when damaged, require a highly regulated healing process. Usually, the prevention of bacterial infection is achieved by using a wound dressing [26]. In this respect, aerogel wound dressings exhibit excellent biocompatibility due to their non-toxicity and low degradability, which provide a barrier against physical, chemical, and biological aggressions. Owing to its large surface area, the aerogel is capable of absorbing fluid without re-leaking and acts as a good thermal insulator [25]. Wound healing formulations using polysaccharides have been extensively studied due to their stability, low toxicity, natural adhesiveness, and good biological performance [7]. In particular, alginate is commonly used in wound healing due to its non-toxicity and biodegradability, which are suitable for the human body [25]. In addition, alginate can offer the positive effects of increasing angiogenesis, enhancing cell migration, and reducing the concentration of proinflammatory cytokines in the wound. Furthermore, the alginate is easily removed because of non-adherence to the wound tissue [7]. Aerogel beads encapsulated with alginate and pectin as the core prepared by prilling and sc-CO_2_ drying have been successfully used for wound healing [26]. In another study, CMC produced by the chemical modification of cellulose has been demonstrated as a good wound dressing due to its flexibility, good exudate absorber, angiogenesis promotion, and autolytic debridement [27]. In addition, the applicability of aerogels can be extended from wound dressing into the drug delivery and antimicrobial fields by incorporating other functionalities. Aerogels of alginate–chitosan fibers have been demonstrated to be effective for wound healing, with a 75% coverage of the wound, which was higher than that of the control, and active inhibition towards *S. aureus* and *K. pneumoniae* [25]. Chitosan is a good candidate for this application because the protonation of the amino groups confers polyelectrolytic features and, hence, antimicrobial properties via interaction with, and the disruption of, the negatively-charged bacterial cell membrane [25]. However, chitosan has a lower absorption capacity for water molecules due to its strong inter/intramolecular hydrogen bonding, thus reducing the intermolecular space for water absorption [61]. Nevertheless, the porous structure and swelling behavior can be improved by crosslinking with other components [57]. For instance, chitosan has been chemically crosslinked with epichlorohydrin and ionically crosslinked with itaconic acid to form a polyelectrolyte complex, thus improving its wound healing properties and having a significant impact on the thermal properties and swelling behavior. The evaluation of the effectiveness of polysaccharide aerogels for wound treatment applications includes the cytotoxicity and antimicrobial properties, as shown in Figure 12. Their negligible toxicity, antibacterial performance on *E. coli* and *C. glutamicum*, and prospective wound healing matrix are demonstrated in this study [61].

**Figure 13:**
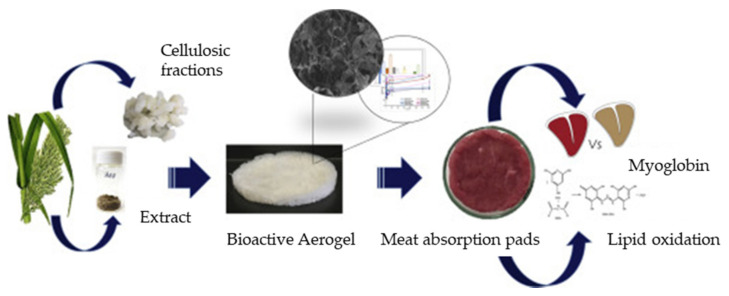
Schematic representation of a cellulose-based aerogel extracted from A. donox biomass for food packaging (antioxidant effect on meat). Reproduced from reference [132] with permission. Copyright 2020 Elsevier.

Presently, there is increasing demand for more environmentally friendly and sustainable food packaging materials from natural sources to replace the depleted fossil-sourced polymers. In food packaging, temperature and humidity are important conditions that can influence the food quality. Therefore, the packaging material should have exceptional insulating properties to ensure a temperature control barrier towards moisture, lipids and oils, volatiles, and gases [40]. At the same time, it should maintain the food quality by incorporating antioxidants that can improve the nutritional value of the packaged product [132]. In addition, the food packaging should have moisture control elements that can absorb the excess liquids in the bioactive pads that are used for meat packaging. Bioactive substances such as citric acid and sodium bicarbonate are also added in order to improve the antimicrobial properties of these packaging pads. The packaging should also be flexible, flavorless, tasteless, odorless, transparent, and colorless [38]. The utilization of aerogel as a packaging material is favored due to its high surface area and the availability of functional groups for interactions with the medium in order to maintain the physical structure. Various types of cellulose have various absorption capacities, as indicated by De Oliveria et al. who observed that the highest water absorption capacity is for oat cellulose nanocrystal aerogel as compared to rice or eucalyptus cellulose nanocrystal aerogels [41]. This is due to the lower pore size and, hence, lower water absorption rate of the eucalyptus cellulose nanocrystal aerogel. In addition, eucalyptus cellulose nanocrystal aerogel has a high relative crystallinity that creates a barrier effect in the polymer matrix, thus causing low diffusion of water between the polymer chains. Other advantages such as the renewability, high surface-area-to-volume ratio, and biocompatibility of cellulose are the major reasons for its promising applicability to food packaging [63]. Polysaccharides are used in food packaging because they are edible and have good film-forming properties. Moreover, they can be molded into any size and shape due to the presence of divalent ions that can undergo gelation with changes in the temperature and pH [37]. Moreover, their high nutritional value, functionality, and low price make them suitable alternatives for use as packaging materials; hence, a number of studies have been conducted in the application of polysaccharides as bio-based packaging material [41]. As noted above, the introduction of metal ions into the aerogel can improve its antimicrobial properties for use in various applications. Nanofillers such as titanium dioxide embedded in a pectin matrix also provide high antimicrobial efficiency, which can prevent food contamination [37,41]. Due to the high porosity of the alginate aerogel, metal ions such as Ca, Ba, Cu, Ni and Zn have been introduced as antimicrobial agents for food packaging applications [38]. However, the various types of metal ion have distinct effects upon the various types of bacteria. For instance, one study has shown that the ZnO nanoparticle provides superior antibacterial activity for *S. aureus* compared to MgO or CaO. The difference is attributed to the interfacial potential difference between the metal ions and the bacteria [133]. The antimicrobial properties of food packaging also arise due to the presence of superoxide, which contains free negatively charged species and positively charged metal ions that can penetrate the bacterial cell wall and cause lipid, protein, and DNA damage, leading to bacteria death. Finally, the taste and appeal are important considerations for food packaging [39]. Figure 13 shows the application of cellulose extracted from *A. donax* biomass as a bioactive superabsorbent aerogel for use in meat absorption pads in food packaging. The extract was incorporated into the aerogel due to its good water absorption capacity, higher antioxidant potential, lower production cost, and lower environmental impact. The in vitro release and β-carotene bleaching inhibition study demonstrated reduced color loss and lipid oxidation of the meat upon refrigerated storage [132].

**Figure 14:**
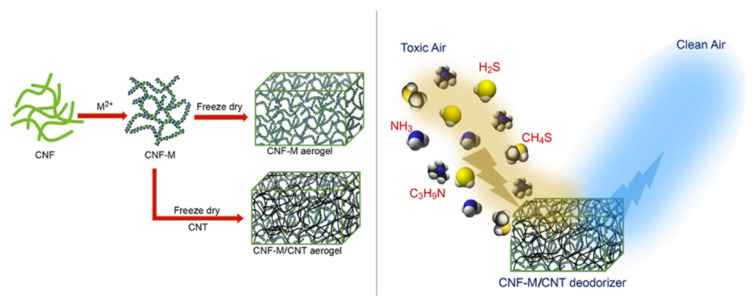
Schematic representation of Cu(II)/cellulose nanofibers/carbon nanotubes for the elimination of toxic gases (H_2_S, NH_3_, C_3_H_9_N, and CH_4_S) from air. Reproduced from reference [94] with permission. Copyright 2020 Elsevier.

Air filtration is an effective way to improve air pollution by using various fiber and composite filter materials. Although non-environmentally friendly materials such as polyacrylonitrile or amines are generally used, the aerogels are promising alternatives due to their continuous 3-D structures, adjustable densities, high specific surface areas, high porosities, and interconnective networks that favor the gas–solid interaction. Thus, aerogel filters with the desired air permeability and efficiency can be fabricated by the accurate control of the nanostructure assembly. Several studies have been performed using cellulose, chitosan, starch, lignin, alginate, and konjac glucomannan as the air filtration material [117]. The filtration performance of the aerogels is evaluated in terms of their filtration efficiency and filtration resistance. Among the harmful and toxic gasses that can be absorbed from the air are carbon monoxide (CO), sulfur dioxide (SO_2_), ammonia (NH_3_), nitrogen oxide (NO_X_) and volatile organic compounds (VOCs). The study has shown that the use of wheat straw aerogel for the removal of particulate matter (particle sizes ≥ 0.3 µm) can provide a filtration efficiency of 93.54%, along with a filtration resistance of 29 Pa and an air permeability of 271.42 L/s·m^2^ [117]. Unfortunately, there are some limitations to the use of polysaccharide aerogels in air filtration such as poor mechanical and hydrophobic properties [113,130]. Nevertheless, the microstructure, and, hence, the mechanical properties of the aerogel can be enhanced via the cross-linking process with polyethylamine, graphene oxide, or amino silane [16]. Thus, their porosity, pore size, cell wall constituents, thicknesses, and macroscopic shapes can be adjusted. As shown in Figure 14, the removal of toxic gases was achieved using oxidized cellulose nanofibril-transition metal divalent cations (M^2+^)/multiwalled carbon nanotubes. This newly developed adsorbent is eco-friendly, ultra-light (5−10 mg/cm^3^), highly porous (99.17%), industry friendly, and is effective for deodorization by the removal of major malodorous gases such as ammonia, amine, hydrogen sulfide, and mercaptans from the air. This is achieved via the coordination of the Cu^2+^ ions with the −OH groups of the cellulose, which provides spaces for the absorption of the odorous molecules. The malodorous gases were successfully separated from 9 l of air via the processes of chemisorption and physisorption such that completely clean air was obtained. Moreover, the removal process worked reliably over a temperature range of 0 to 40 °C [94].

In the CO_2_ adsorption process, the surface chemical structure of the aerogel can be modified via amine functionalization to increase the adsorption process. For instance, an amine functionalized cellulose aerogel was prepared by grafting 3-aminopropyltriethoxysilane onto cellulose gel to increase the nanopores after pore space blockage, thus providing an adsorption capacity of 1.20 mmol/g with dry 1% CO_2_ [15] along with great stability over 20 adsorption–desorption cycles. The reusability of an aerogel for CO_2_ adsorption was also demonstrated in a study by Verma et al., who showed that a cellulose-crosslinked polyethyleneimine aerogel was able to absorb CO_2_ for up to 10 cycles [16]. However, the use of natural polysaccharides is still new, particularly in biomedical applications. To meet the requirements of the final application, the properties of the aerogels should be improved in order to achieve non-toxicity, sustainability, biocompatibility, and biodegradability. This can be achieved by combining polysaccharides with other components that can extend the material application [134]. In addition, the hybrid aerogels with molecular-aggregate level compositions exhibit unique, folded microstructures and enhanced mechanical properties [135].

## 6. Limitations, Conclusions, and Outlook

The literature survey clearly demonstrates the potential applicability of polysaccharide aerogels due to their favorable properties compared to those of the presently available synthetic aerogels. However, the hydrophilic nature conferred on the polysaccharides by the presence of −OH groups results in poor mechanical and thermal properties that need to be addressed. Nevertheless, the easy hybridization or cross-linking with other materials renders the aerogels suitable for a vast range of applications including in the biomedical field, as efficient adsorbents or air filtration systems, and in the food packaging industry. As widely reported, various nanofillers have been employed to upgrade the final properties of the aerogels, including carbon, inorganic materials, metal ions (TiO_2_, SiO_2_, or Ag), or chemical reagents such as diisocyanate, aldehyde, and *N*,*N*′-methylenebis(acrylamide).

Although a wide range of studies have been performed on the polysaccharide aerogels, some limitations should be considered in order to reduce the existing limitations for their potential application in industry. These include the complex fabrication process, high manufacturing costs, and high failure rates. Considering the natural support that the polysaccharides can offer, they should be further explored for increased cost effectiveness, non-toxicity, and abundant availability. Nevertheless, the requirements of a well-defined structure, definite bioactivity, biocompatibility, security, and appropriate physico-chemical properties must be satisfied.

Even though the production of natural polysaccharide aerogels from cellulose nanofibrils and bacterial cellulose resulted in the formation of effective adsorbents, their production process is generally complicated and incurs additional costs. Hence, a thorough study on the techniques, equipment, methods, and process conditions during aerogel preparation could be resulted in polysaccharide aerogels with outstanding characteristics. Moreover, the development of a regenerative (i.e., reusable) adsorbent could offer a more economical, less time consuming, and energy effective solution. In particular, the development of polysaccharide aerogels for use as adsorbents should satisfy the criterion of recyclability in order to compete with currently commercial adsorbents such as silica.

In addition, the use of chemical reagents during crosslinking with polysaccharides is not a good environmental solution and is unsuitable for biomedical applications. Thus, it is important to select suitable chemical reagents for the crosslinking process. Although some studies have introduced graphene–polysaccharide aerogel for its high absorption capacity and outstanding recyclability performance, its use is still restricted due to the large amount of chemicals used and acid waste generated. Besides, it does not exhibit practical reusability, especially as an adsorbent.

The most critical stage of aerogel synthesis is drying, and two widely used methods are sc-CO_2_ and freeze drying. The drying phase influences the morphology, porosity, and structural integrity of the aerogel. The sc-CO_2_ drying method can provide a strong, porous structure; however, it requires conditions of very high pressure and temperature, thus making it an energy intensive, dangerous, and expensive process. These conditions are not generally favorable for industrial application. Moreover, the freeze drying method is also time-consuming, during which the network structure is easily destroyed. To overcome these limitations, it is crucial to develop enhanced process conditions, such as fabrication under ambient pressure without causing structural changes. Surface modification should also be taken into account for improving the efficiency of the drying process. However, most of the surface modification occurs microscopically and involves a large pore size that can destroy the homogeneity and integrity of the aerogel.

The applications of polysaccharide aerogels can be expanded by converting them into various forms such as sheets or blocks rather than droplets or fibers. It is also critical to study polysaccharide aerogels in the form of monoliths during the sc-CO_2_ drying process. This is because shrinkage may occur due to capillary stress during the intermediate stages. This eventually leads to the easy collapse of the pores under high stress during mechanical testing. Therefore, research into improving the mechanical properties of the aerogel monolith should be pursued. Further, functional modification could provide colorful aerogel fibers, phase-change fibers, conductive fibers, or hydrophobic fibers for textile applications.

In conclusion, improvements are needed in order to enhance the prospects of the polysaccharide aerogels with respect to commercialization and industrial application as an alternative to available commercialized silica aerogels. The requisite improvements include the large scale, low-cost fabrication of polysaccharide aerogels with outstanding physical and mechanical properties. In addition, the successive regeneration and reuse of adsorbents is an important consideration for practical applications.

## Figures and Tables

**Table 1:** The physical properties of microparticle-sized polysaccharides and aerogels.

Polysaccharides	Surface Area (m^2^/g)	Pore Size (%)	Density (g/cm^3^)	Reference
Starch	52.6–59.7	-	0.05–0.29	[6]
Cellulose	800	-	0.004–0.04	[18]
Cellulose/carbon	5.07–47.3	76–98.8	-	[18]
Aerocellulose	290	92	0.125	[60]
Cellulose (Rice Straw)	178.8	99.8	0.0022–0.024	[63]
Cellulose/MNCF	-	99.5	0.0072	[63]
Cellulose/PAE/MNFC	-	98.4	0.002–0.024	[63]
Chitosan	25.7–47.9	-	-	[64]
Chitosan	112	91	1.53	[65]

**Table 2:** The crosslinking conditions of various polysaccharides (2020).

Polysaccharides	Organic/Inorganic	Drying Methods	References
Fiber-shaped template (PANI@PP@GPN) 1,2-Epoxypropane Carboxymethyl cellulose	Ti	FD	[90]
	SiO_2_–ZrO_2_	sc-CO_2_	[91]
	Graphene oxide	FD	[92]
Cellulose and carbon fibers Cellulose nanofibers and carbon nanotubes	Reduced graphene oxide	FD	[93]
	copper (II)	FD	[94]
Bacterial cellulose Biomass cellulose and carbon fibers	Polyaniline nanocomposite; sodium dodecylbenzene-sulfonate/sodium dodecyl sulfate	FD	[11]
	Silver nanoparticle/polyaniline	FD	[29]
	graphene oxide	FD	
	Reduced graphene oxide	FD	[93]
Cellulose Bacterial cellulose/chitosan composite aerogel	Metalorganic framework		
	Metal–organic framework	FD	[95]

**Table 3:** Polysaccharide aerogels and their applications.

Polysaccharides	Characteristics	Applications	References
Bacterial cellulose/polyaniline nanocomposite	Surface and pore volume diffusion simultaneously exist in the adsorption process.	Removal of hexavalent chromium	[11]
Amine grafted cellulose aerogel	Interconnective networks that favor interactions	CO_2_ separation	[15]
Twisted carbon fiber (TCF)	Good recyclability	Adsorbent	[17]
Raw cotton	Can be regenerated many times	Oil/organic adsorption	[17]
Wood cellulose aerogel	Ultra-low thermal conductivity	Thermal insulation	[29]
Pectin	Mechanical, thermal, and antimicrobial properties are improved	Food packaging	[37]
Biomass (wheat straw, gum, gelatin)	Excellent efficiency 0.35	Sound absorption	[43]
Nanofibril chitin	Mesoporous aerogel structure	Renewable catalysts	[53]
Chitosan aerogel	Excellent swelling behavior	Wound dressings	[61]
Germinated and non-germinated wheat starch	High water absorption, high degradation temperature	Food packaging	[73]
Nanocellulose/graphene aerogel	Improved hydrophobicity	Oil treatment/absorption	[79]
Nanocellulose/silver nanoparticles	Improved stability and dispersibility of the metal nanoparticles.	Catalytic discoloration	[80]
Fiber-shaped aerogel templates (glycerol-functionalized PAN nanofibers (GPN))	High porosity, large void space, combined hydrophilic and lipophilic characteristics	Supercapacitors	[90]
Copper (II)-cellulose nanofibers and carbon nanotubes	Easy production for industrial applications. Suitable for a wide range of temperatures	Air filtration	[94]
Carrageenan/cellulose nanocrystal/silver nanoparticles	Antibacterial properties	Wound dressing	[95]
Cellulose triacetate	Pores structure	Drug delivery system	[100]
Cellulose/chitosan	Flexible bulk	Thermal insulation in microwave ovens	[110]
Chitosan/montmorillonite/carbon nanotube composite aerogel	Excellent flame retardancy	Fire-resistance	[112]
Cellulose	High porosity, 94%	Flame retardancy	[114]
Bamboo fungus tube-like aerogel	Good microstructure and morphology	Oil/water separation	[115]
Protein−polysaccharide conjugates	Increased viscosity and increased elastic modulus	Oil removal	[116]
KGM/gelatin/starch/wheat straw/okara	Improved functional properties	Air filtration	[117]
